# Prognosis of liver transplantation: Does postoperative ileus matter?

**DOI:** 10.1186/s12876-021-02026-7

**Published:** 2021-11-25

**Authors:** Ruiping Bai, Rui An, Kunyu Han, Mengwen Xue, Simei Zhang, Xin Shen, Shaohua Zheng

**Affiliations:** 1 Department of Anaesthesiology, The First Affiliated Hospital of Xi’an Jiaotong University, Shaanxi Xi’an, China; 2 Department of Hepatobiliary Surgery, The First Affiliated Hospital of Xi’an Jiaotong University, Xi’an, China

**Keywords:** Liver transplantation, Postoperative ileus, MELD score, Child–Pugh score

## Abstract

**Background:**

Nowadays, liver transplantation has become a main therapy for end-stage liver disease. However, studies show that there are high mortality and severe complications after liver transplantation. Although gastrointestinal dysfunction is a common and major complication after liver transplantation, there was rarely relative research. This study aims to elucidate the factors about ileus after liver transplantation and patients’ survival.

**Methods:**

We collected and analyzed the data (n = 318, 2016–2019) from the First Affiliated Hospital of Xi’an Jiaotong University. After excluding cases, a total of 293 patients were included for this study. The subjects were divided into a non-ileus group and an ileus group. We reviewed 38 variables (including preoperative, operative and postoperative relative factors). Additionally, other complications after liver transplantation and survival data were compared between two groups.

**Results:**

Of the 293 patients, 23.2% (n = 68) experienced postoperative ileus. Ileus patients were not different with non-ileus patients in preoperative, operative and postoperative factors. HBV-positive patients with ileus had a lower MELD score (*P* = 0.025), and lower postoperative total bilirubin was correlated with ileus (*P* = 0.049). Besides, Child–Pugh score of HCC patients with ileus was low (*P* = 0.029). The complications after liver transplantation were not different between two groups. Compared with the patients without ileus, the patients with ileus had a higher mortality rate.

**Conclusion:**

According to our research, ileus-patients had a lower 1-year survival rates. The preoperative MELD score and postoperative total bilirubin of HBV-positive patients with ileus were lower, and Child–Pugh score of HCC patients with ileus was also lower.

## Background

Liver disease accounts for approximately 2 million deaths every year worldwide [1]. Apart from cirrhosis and hepatocellular carcinoma due to viral hepatitis as well as alchohol, non-alchoholic fatty liver disease and drug induced hepatitis continue to increase as a main cause of acute liver injury. Liver diseases were estimated to become the 12th leading cause of mortality by 2020 [2]. Liver transplantation (LT) becomes a major therapy for liver diseases, especially the end-stage liver disease [1, 3, 4]. However, the needs of liver transplantation far exceed the supply. Current liver transplantation rates were less than 10% needs of organ transplantation [1]. Although the survival rate of liver transplantation has improved greatly in recent years, there are still many complications that affect prognosis and life span [5].

The clinical researches about cardiovascular disease, acute kidney injury and thrombosis after LT are most common, and gastrointestinal dysfunction is an ordinary complication after surgery, but postoperative ileus (POI) after LT is rare [6–8]. Postoperative ileus is a common complication following especially open abdominal surgery. Preoperative malnutrition, operative procedure, anesthesia and postoperative managements may increase the risk of gastrointestinal dysfunction [9]. According to clinical experience, once the gastrointestinal function is abnormal, the recovery of LT patients is delayed, even may affect survival rate of patients. Our study is designed to identify risk factors, obtain possible predictive factors and the effects of ileus to survival rate after LT patients.

## Methods

### Data collection

We collected the data from the First Affiliated Hospital of Xi’an Jiaotong University from January 2016 to March 2019 all recipients that underwent orthotopic liver transplantation (OLT). All of the liver grafts were from cardiac-dead donors. We excluded the candidates who were in gastrointestinal dysfunction before surgery, needed enteral feeding or nasogastric tube after surgery, homeostasis disturbance, secondary liver transplantation, multiple organ dysfunction syndrome (MODS) before surgery, critically ill after surgery (included extended immobility or narcotic use) and incomplete data. The duct-to-duct biliary reconstruction was performed for every patient, and there were no any cases of biliodigestive anastomosis. Data elements included preoperative, operative and postoperative factors. Additionally, patients were classified as postoperative ileus when the time of the first passage of flatus and the first defecation was over 72 h, or normal. In our study, all ileus-patients were paralytic ileus without requiring reoperation for adhesiolysis, and there were no patients with mechanical ileus.

### Follow up

In view of regular check-up of post-LT patients, we used the inhospital or outhospital numbers, which are unique to each patient, to obtain patients’ outcome in this study. The single endpoint of the study was all-cause mortality. Observations were stopped at the date of one year after surgery. Finally, 293 patients finished follow up.

### Statistic analysis

The patients were categorized into 2 groups according to postoperative ileus. Continuous variables were summarized as means ± standard deviation or median and interquartile ranges, whereas frequencies and percentages were used for categorical variables. Student’s t-test or Mann–Whitney U-test for continuous independent variables, the Pearson ${chi}^{2}$ test or Fisher’s exact test was used to compare qualitative variables. The log-rank test (Mantel-Cox) was used to compare group survival curves. Statistical significance for all analyses was determined at *P* < 0.05. All analyses were undertaken by using Statistical Package for Social Sciences (SPSS) software, version 25.0 (IBM SPSS, Armonk, NY, USA).

## Results

### Patient’s characteristics of eligible transplanted cohort

There were 293 patients who were eligible and finished the follow up. The mean age of the transplanted population was 48 (40.00–55.00) and 76.5% were males. The most common atiology of liver diseases were chronic hepatitis B (46.4%), primary hepatic carcinoma (31.4%), and chronic hepatitis C (4.1%). Less common atiology included alcohol liver disease (7 patients), autoimmune hepatitis (7 patients), cholestatic hepatitis (7 patient), and NASH (31 patients) (Table 1).

**Table 1:** Preoperative and operative relative factors

	Over all (n = 293)	Ileus (n = 68)	Non-ileus (n = 225)	*P* value
Preoperative characteristics				
Age (years)	48.0 (40.00–55.00)	48.0 (37.00–54.75)	47.9 ± 10.08	0.676
Male	224.0 (76.50%)	53.0 (77.90%)	171.0 (76.00%)	0.741
BMI (kg/m^2^)	22.5 (20.76–24.47)	22.6 ± 3.19	22.3 (20.76–24.39)	0.952
History of abdominal surgery	75.0 (25.60%)	20.0 (29.40%)	55.0 (24.40%)	0.411
Hepatic encephalopathy	33.0 (11.30%)	7.0 (10.30%)	26.0 (11.60%)	0.773
Portal hypertension	197.0 (67.20%)	47.0 (69.10%)	150.0 (66.70%)	0.706
Pleural fluid or ascites	143.0 (48.80%)	31.0 (45.60%)	112.0 (49.80%)	0.545
Child–Pugh	10.0 (9.00–11.00)	10.0 (8.00–11.00)	10.0 (9.00–11.00)	0.289
MELD score	15.0 (11.00–23.00)	14.5 (10.00–19.75)	16.0 (11.50–23.00)	0.058
Albumin (g/L)	36.8 (32.90–42.20)	36.7 ± 6.57	37.0 (32.95–42.25)	0.432
Total bilirubin (umol/L)	44.8 (25.05–135.40)	44.8 (22.30–98.40)	44.8 (26.10–168.35)	0.363
Serum creatinine (umol/L)	56.0 (46.00–67.00)	57.0 (45.50–75.00)	55.0 (46.00–66.00)	0.370
Preoperative lactic acid	1.5 (1.10–1.90)	1.5 (1.00–2.00)	1.4 (1.10–1.90)	0.725
Atiology of liver disease				
Chronic hepatitis B	136.0 (46.40%)	34.0 (50.00%)	102.0 (45.30%)	0.499
Primary hepatic carcinoma	92.0 (31.40%)	18.0 (26.50%)	74.0 (32.90%)	0.318
Chronic hepatitis C	12.0 (4.10%)	1.0 (1.50%)	11.0 (4.90%)	0.370
Alcoholic hepatitis	7.0 (2.40%)	1.0 (1.50%)	6.0 (2.70%)	0.910
Autoimmune hepatitis	7.0 (2.40%)	4.0 (5.90%)	3.0 (1.30%)	0.089
Cholestatic Cirrhosis	7.0 (2.40%)	1.0 (1.50%)	6.0 (2.70%)	0.910
Other	31.0 (10.60%)	9.0 (13.20%)	22.0 (9.80%)	0.417
Intraoperative factors				
Operation time (min)	365.0 (330.00–420.00)	377.0 ± 81.52	367.0 (330.00–420.00)	0.668
Anhepatic phase (min)	50.0 (45.00–57.00)	51.4 ± 11.59	51.0 (45.00–57.00)	0.578
Portal vein clamping time (min)	58.0 (52.50–65.00)	57.0 (52.00–65.50)	58.0 (53.00–65.00)	0.546
Blood loss during operation (ml)	1000.0 (650–2000)	1000.0 (625–1600)	1000.0 (650–2000)	0.661
Total infusion fluid (ml)	5660.0 (4735–6690)	5750.7 ± 1653.65	5700.0 (4780–6710)	0.385
Intraoperative RBC transfusion (units)	8.0 (4.00–12.00)	8.0 (4.00–11.75)	8.0 (6.00–12.00)	0.167
Intraoperative cryoprecipitate transfusion(units)	1200.0 (800–1600)	1000.0 (800.00–1550.00)	1200.0 (800.00–1600.00)	0.177
Retention time of tracheal tube (hours)	7.2 (5.25–9.63)	7.3 (5.50–9.44)	7.2 (5.00–9.88)	0.917
Length of SICU (days)	6.0 (5.00–10.00)	6.0 (5.00–9.50)	7.0 (5.00–10.00)	0.394
Length of hospital stay (days)	19.0 (15.00–24.50)	19.5 (15.00–23.75)	19.0 (15.00–26.00)	0.947
Anesthesia factors				
Propofol (mg)	1500.0 (1300.00–1820.00)	1500.0 (1200.00–1930.00)	1500.0 (1300.00–1800.00)	0.826
Sufentanil (ug)	30.0 (30.00–40.00)	30.0 (30.00–40.00)	30.0 (30.00–40.00)	0.101
Renifentanil (ug)	3000.0 (2517–3800)	3000.0 (2400–3956)	3000.0 (2600–3600)	0.773
Sevoflurane (ml)	60.0 (50.00–80.00)	60.0 (50.00–70.00)	60.0 (50.00–80.00)	0.796
Dexmedetomidine (ug)	120.0 (100.00–180.00)	111.0 (100.00–150.00)	120.0 (85.00–180.00)	0.881
Etomidate (mg)	14.0 (10.00–16.00)	14.0 (11.25–16.00)	14.00 (10.00–16.00)	0.572

### Preoperative risk factors of postoperative ileus

The study demonstrated that age, male, BMI (body mass index), history of abdominal surgery, hepatic encephalopathy, portal hypertension, Model for End-Stage Liver Disease (MELD score), platelet counts, albumin, total bilirubin, serum creatinine, lactic acid and atiology of liver disease were not significantly different between groups (Table 1).

Table 2 shows the results of risk factors for HBV-positive patients with and without ileus. Except for MELD score (*P* = 0.025), no significant differences were observed between patients with and without ileus for other factors analyzed.

**Table 2:** Preoperative and operative relative factors of patients with HBV

	Over all (n = 192)	Ileus (n = 47)	Non-ileus (n = 145)	*P* value
Preoperative characteristics				
Age (years)	45.4 ± 9.17	45.7 ± 9.25	46.5 ± 9.41	0.633
Male	158.0 (82.30%)	40.0 (85.10%)	118.0 (81.40%)	0.561
BMI (kg/m^2^)	22.2 (20.76–24.22)	22.8 ± 2.94	22.328 (20.76–24.39)	0.772
History of abdominal surgery	49.0 (25.50%)	14.0 (29.80%)	35.0 (24.10%)	0.440
Hepatic encephalopathy	21.0 (10.90%)	4.0 (8.50%)	17.0 (11.70%)	0.540
Portal hypertension	135.0 (70.30%)	34.0 (72.30%)	101.0 (69.70%)	0.726
Pleural fluid or ascites	89.0 (46.40%)	20.0 (42.60%)	69.0 (47.60%)	0.548
Child–Pugh	10.0 (9.00–12.00)	9.4 ± 2.06	10.0 (9.00–11.50)	0.055
MELD score	16.0 (12.00–24.00)	13.0 (9.00–19.00)	16.0 (11.00–23.00)	***0.025***
Albumin (g/L)	35.7 (31.80–41.48)	37.0 ± 6.90	37.1 (32.90–43.30)	0.536
Total bilirubin (umol/L)	45.9 (25.93–127.05)	32.7 (21.30–98.50)	46.8 (26.05–126.70)	0.140
Serum creatinine (umol/L)	57.0 (46.00–71.75)	57.0 (44.00–76.00)	55.0 (46.00–66.00)	0.499
Preoperative lactic acid	1.5 (1.10–1.88)	1.5 (1.00–1.80)	1.4 (1.10–1.80)	0.722
Intraoperative factors				
Operation time (min)	390.0 (330.00–420.00)	371.4 ± 93.24	365.0 (330.00–420.00)	0.401
Anhepatic phase (min)	50.0 (45.00–57.00)	50.0 (45.00–59.00)	51.0 (45.00–57.50)	0.862
Portal vein clamping time (min)	58.50 (53.00–65.75)	58.0 (53.00–67.00)	59.0 (52.50–65.50)	0.930
Blood loss during operation (ml)	1200.0 (800–2000)	1000.0 (600–1600)	1200.0 (800–2000)	0.446
Total infusion fluid (ml)	5830.0 (5032–6820)	5410.0 (4490–6524)	5710.0 (4690–6725)	0.210
Intraoperative RBC transfusion (units)	8.0 (6.00–12.00)	6.0 (4.00–12.00)	8.0 (4.50–12.00)	0.167
Intraoperative cryoprecipitate transfusion (units)	1400.0 (1000–1600)	1000.0 (800.00–1600.00)	1200.0 (1000–1600)	0.245
Retention time of tracheal tube (hours)	7.0 (5.00–9.50)	6.3 (5.25–11.00)	7.3 (5.00–9.63)	0.328
Length of SICU (days)	6.0 (4.00–10.00)	6.0 (4.00–8.00)	7.0 (5.00–10.00)	0.213
Length of hospital stay (days)	18.0 (14.00–23.00)	18.0 (14.00–23.00)	18.0 (15.00–26.00)	0.721
Anesthesia factors				
Propofol (mg)	1500.0 (1400–1885)	1609.6 ± 452.87	1500.0 (1300–1990)	0.985
Sufentanil (ug)	30.0 (30.00–40.00)	30.0 (25.00–40.00)	30.0 (30.00–40.00)	0.209
Renifentanil (ug)	3000.0 (2725–4000)	3219.6 ± 996.15	3000.0 (2600–4000)	0.868
Sevoflurane (ml)	60.0 (50.00–80.00)	60.0 (50.00–80.00)	60.0 (50.00–70.00)	0.355
Dexmedetomidine (ug)	116.0 (80.25–186.75)	100.0 (100.00–150.00)	120.0 (80.00–189.50)	0.627
Etomidate (mg)	14.0 (10.00–16.00)	14.0 (12.00–16.00)	14.0 (10.00–16.00)	0.288

Bold italics means P < 0.05

There were not different between with and without ileus of HCC (hepatocellular carcinoma) patients for intraoperative risk factors, except for Child–Pugh score (*P* = 0.029; Table 3).

**Table 3:** Preoperative and operative relative factors of patients with HCC

	Over all (n = 92)	Ileus (n = 18)	Non-ileus (n = 74)	*P* value
Preoperative characteristics				
Age (years)	50.1 ± 9.33	50.2 ± 8.83	50.0 ± 9.51	0.959
Male	77.0 (83.70%)	18.0 (100.00%)	59.0 (79.70%)	0.083
BMI (kg/m^2^)	23.3 ± 3.33	23.4 ± 2.75	23.2 ± 3.47	0.832
History of abdominal surgery	17.0 (18.50%)	4.0 (22.20%)	13.0 (17.60%)	0.906
Hepatic encephalopathy	3.0 (3.30%)	0 (0.0%)	3.0 (4.10%)	1.000
Portal hypertension	47.0 (51.10%)	10.0 (55.60%)	37.0 (50.00%)	0.672
Pleural fluid or ascites	30.0 (32.60%)	6.0 (33.30%)	24.0 (32.40%)	0.942
Child–Pugh	9.0 (8.00–11.00)	8.0 (6.00–10.25)	9.0 (8.00–11.00)	***0.029***
MELD score	12.0 (9.00–18.00)	11.6 ± 4.46	12.0 (10.00–19.25)	0.081
Albumin (g/L)	39.7 ± 5.97	36.0 ± 7.06	39.9 ± 6.12	0.488
Total bilirubin (umol/L)	34.9 (19.47–77.48)	30.9 (15.48–68.48)	35.5 (19.63–87.63)	0.425
Serum creatinine (umol/L)	55.0 (46.00–64.75)	63.1 ± 26.72	54.5 (46.00–63.25)	0.394
Preoperative lactic acid	1.3 (1.03–1.70)	1.4 (1.00–1.80)	1.3 (1.08–1.70)	0.657
Intraoperative factors				
Operation time (min)	360.0 (300.00–420.00)	369.2 ± 71.71	360.0 (300.00–412.50)	0.726
Anhepatic phase (min)	50.0 (45.00–57.75)	52.4 ± 12.67	50.0 (45.00–57.25)	0.976
Portal vein clamping time (min)	58.5 (52.75–65.25)	58.0 (51.00–67.00)	59.0 (53.00–65.00)	0.774
Blood loss during operation (ml)	800.0 (500.00–1200.00)	800.0 (575.00–1050.00)	800.0 (500.00–1225.00)	0.832
Total infusion fluid (ml)	4915.0 (4285–5960)	4656.2 ± 1190.72	5070.0 (4308–6145)	0.080
Intraoperative RBC transfusion (units)	5.0 (2.00–9.50)	4.0 (1.50–6.50)	6.0 (2.00–10.00)	0.181
Intraoperative cryoprecipitate transfusion (units)	800.0 (600.00–1200.00)	800.0 (750.00–1000.00)	800.0 (600.00–1200.00)	0.420
Retention time of tracheal tube (hours)	7.0 (5.00–9.88)	9.2 ± 4.63	6.8 (5.00–9.31)	0.162
Length of SICU (days)	6.0 (5.00–9.00)	6.0 (5.00–8.00)	6.0 (5.00–9.00)	0.714
Length of hospital stay (days)	18.0 (15.00–25.75)	17.5 (14.00–22.50)	19.0 (15.00–26.00)	0.427
Anesthesia factors				
Propofol (mg)	1500.0 (1200–1970)	1522.2 ± 542.93	1500.0 (1300–1909)	0.432
Sufentanil (ug)	30.0 (30.00–40.00)	30.0 (30.00–40.00)	30.0 (30.00–40.00)	0.791
Renifentanil (ug)	3000.0 (2500–3750)	3116.7 ± 1169.84	3000.0 (2500–3650)	0.596
Sevoflurane (ml)	50.0 (50.00–80.00)	50.0 (50.00–62.50)	55.0 (47.50–80.00)	0.920
Dexmedetomidine (ug)	100.0 (100.00–160.00)	100.0 (100.00–162.50)	100.0 (89.25–160.00)	0.783
Etomidate (mg)	14.0 (12.00–16.00)	14.0 (12.00–16.00)	14.0 (11.50–16.00)	0.284

Bold italics means P < 0.05

### Intraoperative and postoperative risk factors of postoperative ileus

Intraoperative factors included duration of surgery, anhepatic phase, blood loss, total infusion fluid, red cell transfusion, cryoprecipitate transfusion and anesthesia (drug doses of propofol, sufentanil, renifentanil, sevoflurane, dexmedetomidine and etomidate) were not different among groups (Table 1). Retention time of tracheal tube, length of Intensive Care Unit of Surgery (SICU), length of hospital-stay, total bilirubin, lactic acid, platelet counts were postoperative possible risk factors. All were not different (Table 4).

**Table 4:** Postoperative characteristics and complications

	Over all (n = 293)	Ileus (n = 68)	Non-ileus (n = 225)	*P* value
Postoperative characteristics				
Total bilirubin (umol/L)	62.1 (40.53–106.05)	58.0 (40.30–94.28)	65.0 (41.13–112.90)	0.315
Lactic acid	2.1 (1.40–3.53)	2.3 (1.40–4.58)	2.0 (1.40–3.23)	0.123
Platelet (× 10^9/L)	48.5 (35.00–73.00)	50.5 (39.00–82.75)	48.0 (32.75–70.25)	0.217
Complications				
Pulmonary infection	6 (2.04%)	3 (4.41%)	3 (1.33%)	0.140
Kidney injury	3 (1.02%)	1 (1.47%)	2 (0.89%)	0.549
Biliary infection	12 (4.10%)	5 (7.35%)	7 (3.11%)	0.158
Vascular complication	5 (1.71%)	2 (2.94%)	3 (1.33%)	0.329
Others	16 (5.46%)	4 (5.88%)	12 (5.33%)	0.770

There were not different between with and without ileus of HBV-positive patients for intraoperative risk factors (Table 2), but postoperative total bilirubin was significantly different between groups (*P* = 0.049; Table 5).

**Table 5:** Postoperative characteristics and complications of patients with HBV

	Over all (n = 192)	Ileus (n = 47)	Non-ileus (n = 145)	*P* value
Postoperative characteristics				
Total bilirubin (umol/L)	62.7 (43.00–106.73)	61.1 ± 30.44	66.4 (44.65–116.58)	***0.049***
Lactic acid	2.2 (1.30–3.53)	2.3 (1.55–4.05)	2.1 (1.30–3.18)	0.186
Platelet (× 10^9/L)	49.5 (35.00–70.75)	47.0 (38.75–75.50)	50.0 (35.00–69.75)	0.820
Complications				
Pulmonary infection	4 (2.08%)	2 (4.26%)	2 (1.38%)	0.252
Kidney injury	3 (1.56%)	1 (2.13%)	2 (1.38%)	0.571
Biliary infection	6 (3.13%)	2 (4.26%)	4 (2.76%)	0.636
Vascular complication	2 (1.04%)	1 (2.13%)	1 (0.69%)	0.431
Others	8 (4.17%)	2 (4.26%)	6 (4.14%)	1.000

Bold italics means P < 0.05

Tables 3 and 6 showed that no significant differences were observed in HCC patients with and without ileus for intraoperative and postoperative risk factors.

**Table 6:** Postoperative characteristics and complications of patients with HCC

	Over all (n = 92)	Ileus (n = 18)	Non-ileus (n = 74)	*P* value
Postoperative characteristics				
Total bilirubin (umol/L)	61.8 (35.15–115.60)	60.3 (36.93–115.13)	62.9 (34.85–115.60)	0.686
Lactic acid	2.0 (1.40–3.40)	2.8 ± 1.62	1.9 (1.40–3.40)	0.401
Platelet (× 10^9/L)	50.5 (35.25–69.50)	57.8 ± 28.36	50.0 (35.00–64.75)	0.638
Complications				
Pulmonary infection	3 (3.26%)	1 (5.56%)	2 (2.70%)	0.484
Kidney injury	2 (2.17%)	1 (5.56%)	1 (1.35%)	0.355
Biliary infection	3 (3.26%)	1 (5.56%)	2 (2.70%)	0.484
Vascular complication	2 (11.80%)	1 (5.56%)	1 (1.35%)	0.355
Others	4 (11.80%)	1 (5.56%)	3 (4.05%)	1.000

### Complications of transplanted cohort

Within the 30 days after surgery, the most common complications were biliary infection or stricture (4.1%), pulmonary infection (2.04%), vascular complication (1.71%), and kidney injury (1.02%). Other complications included acute injection (3 patients), abdominal infection (2 patients), recurrence of hepatocellular carcinoma (5 patients), sepsis (1 patients), coagulation disorders (4 patients), and acute pancreas (1 patient). Between ileus group and non-ileus group were not different (all *P* > 0.05; Table 4). There were no differences in complications between patients with and without ileus of HBV-positive patients (Table 5). Furthermore, no significant differences were observed in HCC (hepatocellular carcinoma) patients with and without ileus (Table 6).

### Overall survival rates of ileus and normal patients

**Fig. 1:**
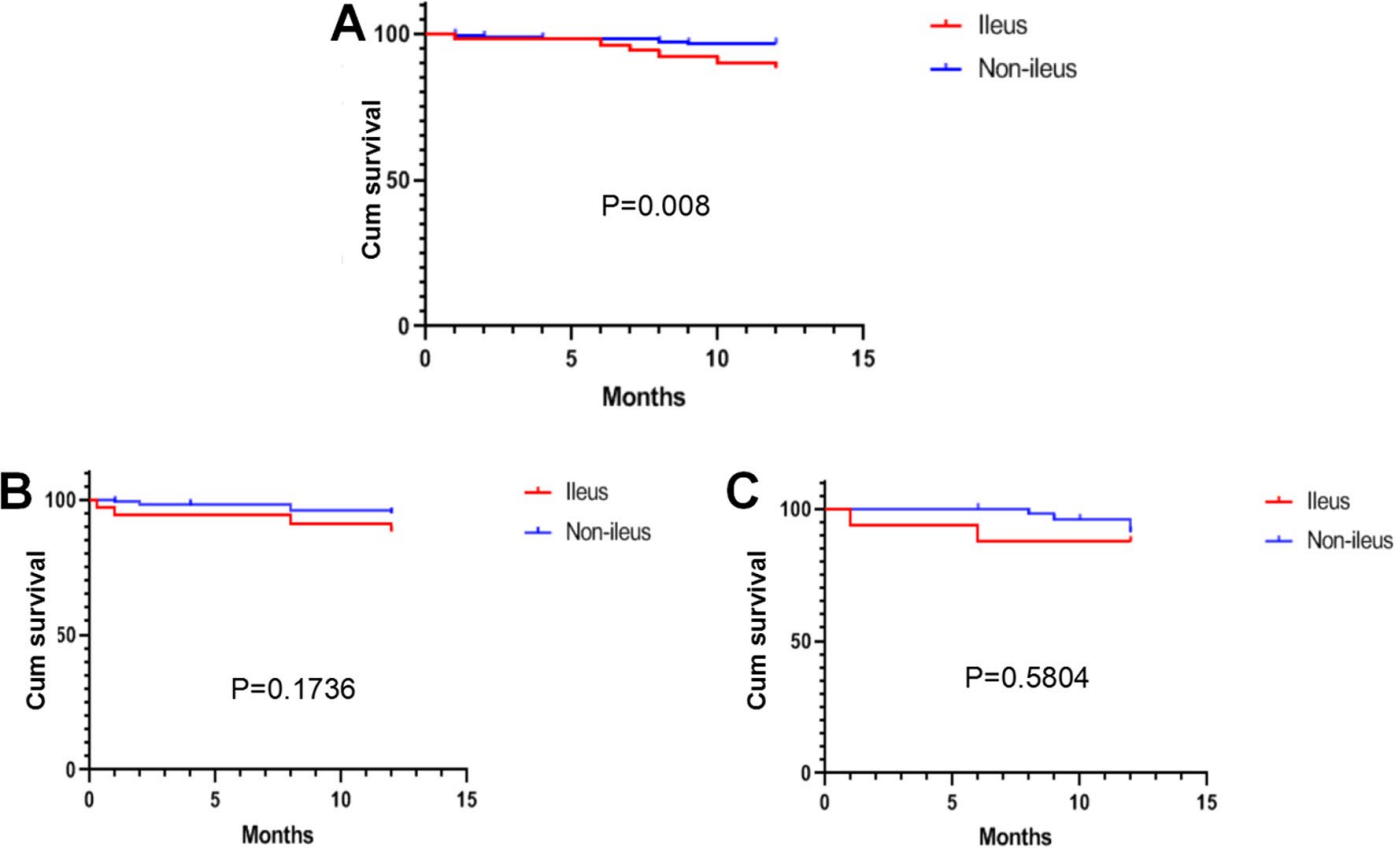
Overall (**A**), HBV-positive (**B**) and HCC (**C**) survival rates of ileus and non-ileus patients

Univariate analysis of 52 patients showed that 1-, 2- and 3-year overall survival rates post-LT were 89%, 89% and 89% in ileus patients, respectively, and 91%, 88% and 88% in non-ileus patients, respectively. The mean survival time was 11.24 months in patients with ileus and 11.76 months in patients without ileus in 1-year overall survival analysis. Kaplan–Meier survival curves showed that the overall survival rate was significantly different (*P* = 0.008) between groups (Fig. 1A).

1-, 2- and 3-year overall survival rates post-LT of HBV-positive patients were 87%, 87% and 87% in ileus patients, respectively, and 91%, 91% and 91% in normal patients, respectively. 1-year overall survival rates post-LT were not different among groups (*P* = 0.174; Fig. 1B).

1-, 2- and 3-year overall survival rates post-LT of HCC patients were 88%, 88% and 88% in ileus patients, respectively, and 93%, 88% and 88% in normal patients, respectively. 1-year overall survival rates post-LT were not different among groups (*P* = 0.580; Fig. 1C).

### Donor's characteristics of ileus and normal patients

Table 7 shows the results of donor's characteristics for patients with and without ileus. There were no significant differences between patients with and without ileus for the factors analyzed.

**Table 7:** The donor's characteristics

	Over all (n = 293)	Ileus (n = 68)	Non-ileus (n = 225)	*P* value
Age (years)	50.0 (39.00–58.00)	52.0 (39.25–59.00)	50.0 (39.00–58.00)	0.439
Male	257.0 (87.70%)	60.0 (88.20%)	197.0 (87.60%)	0.881
BMI (kg/m^2^)	22.5 (20.52–24.31)	22.5 (20.76–24.35)	22.2 ± 3.13	0.502
Warm ischemia time (min)	14.0 (11.00–15.00)	12.5 (10.25–15.00)	14.0 (11.00–15.50)	0.180
Cold ischemia time (hours)	6.0 (5.00–6.00)	6.0 (5.00–6.00)	6.0 (5.00–6.00)	0.527

## Discussion

Liver transplantation is not only a definitive treatment for liver diseases, but also is the second most common solid organ transplantation. Although doctors and researchers take complications of liver transplantation seriously and carry out treatments, POI is ignored by researchers. Postoperative ileus is a common complication after most abdominal surgeries, which is associated with longer hospitalization and increased medical costs [10]. Fluid overload, exogenous opioids, surgical procedure are key mechanisms of POI [11, 12]. Among the 293 patients enrolled in this study with liver diseases who underwent LT, univariate analysis indicated that there was no significant difference in preoperative, intraoperative and postoperative factors between patients with and without POI, apart from the lower MELD score and postoperative total bilirubin in HBV-patients with ileus, and Child-pugh score of HCC patients with ileus was low. Study has reported that goal-directed fluid therapy does not reduce postoperative ileus in gastrointestinal surgery. It is possible that fluid overload is not necessary risk factor [13], which also been proved in our results. Besides, patients with POI had worse overall survival rates than patients without POI.

Model of end-stage liver disease (MELD) and Child–Pugh scores have been widely used to assess the prognosis and predict the outcomes of cirrhotic patients [14]. MELD score is incorporated only 3 objective variables, including total bilirubin, creatinine and INR. Studies has proved that the MELD score system could reduce the mortality in patients waiting for a liver transplantation, and downgrading MELD score can improve the outcomes after liver transplantation in patients with acute-on-chronic hepatitis B liver failure [15, 16]. The Child–Pugh score, based on clinical symptoms of insufficient liver function (ascites/encephalopathy), and laboratory analysis of parameters of liver function (albumin, bilirubin, and PT) can be used to identify low or high-risk patients [17]. And the Child–Pugh score has been proved that it is not only as a predictor of postoperative mortality after portocaval shunt surgery but also predicts mortality risk associated with other major operations [18].The preoperative MELD score and postoperative total bilirubin of HBV-positive patients with ileus were lower than without ileus. And Child–Pugh score of HCC patients with ileus was lower than without ileus. There were no differences in other observed factors and overall survival rates among groups. MELD and Child–Pugh scores are lower, the liver function is better. According to the results, we can get that POI after liver transplantation may predict the recovery of normal liver function. Then, complications after liver transplantation including biliary infection, vascular complication, pulmonary infection, kidney injury were not different in LT patients with ileus and without ileus according to our study. We demonstrated that compared with the patients without ileus, the patients with ileus had a higher mortality rate within one year after surgery, which is consistent with the clinical observation.

Several limitations of this study must be considered. First, this study was retrospective. Second, postoperative ileus was determined based on medical history, and the diagnosis of postoperative ileus lacks objective standards, which needs further research and discussion of professionals, especially ileus after liver transplantation. However, this study indicate that ileus of liver transplantation is a worthy research direction and demands sufficient clinical attention.

## Conclusion

In conclusion, compared with non-ileus patients, we didn’t obtain the risk factors of patients with ileus. Ileus-patients didn’t increase complications after liver transplantation, but decrease post-LT one-year survival rates. But the preoperative MELD score and postoperative total bilirubin of HBV-positive patients with ileus were lower, and Child–Pugh score of HCC patients with ileus was also lower. A future prospective cohort study with larger a sample size should be conducted to confirm these observations, or POI may not be vital in the liver transplantation patients.

## Data Availability

The data that support the findings of this study are available from the corresponding authors.

## Abbreviations

LT: Liver transplantation
POI: Postoperative ileus
OLT: Orthotopic liver transplantation
MODS: Multiple organ dysfunction syndrome
BMI: Body mass index
MELD score: Model for End-Stage Liver Disease
HCC: Hepatocellular carcinoma
SICU: Intensive Care Unit of Surgery
